# Ginsenoside Rb3 Protects Cardiomyocytes against Ischemia-Reperfusion Injury via the Inhibition of JNK-Mediated NF-κB Pathway: A Mouse Cardiomyocyte Model

**DOI:** 10.1371/journal.pone.0103628

**Published:** 2014-08-01

**Authors:** Lijia Ma, Huimin Liu, Zulong Xie, Shuang Yang, Wei Xu, Jingbo Hou, Bo Yu

**Affiliations:** Department of Cardiology, Second Affiliated Hospital of Harbin Medical University, Key Laboratory of Myocardial Ischemia Mechanism and Treatment Ministry of Education, Harbin, Heilongjiang, China; Indiana University School of Medicine, United States of America

## Abstract

Ginsenoside Rb3 is extracted from the plant Panax ginseng and plays important roles in cardiovascular diseases, including myocardial ischemia-reperfusion (I/R) injury. NF-κB is an important transcription factor involved in I/R injury. However, the underlying mechanism of ginsenoside Rb3 in myocardial I/R injury remains poorly understood. In the current study, a model of myocardial I/R injury was induced via oxygen and glucose deprivation (OGD) followed by reperfusion (OGD-Rep) in mouse cardiac myoblast H9c2 cells. Our data demonstrate that ginsenoside Rb3 suppresses OGD-Rep-induced cell apoptosis by the suppression of ROS generation. By detecting the NF-κB signaling pathway, we discover that the protective effect of ginsenoside Rb3 on the OGD-Rep injury is closely related to the inhibition of NF-κB activity. Ginsenoside Rb3 inhibits the upregulation of phospho-IκB-α and nuclear translocation of NF-κB subunit p65 which are induced by ORD-Rep injury. In addition, the extract also inhibits the OGD-Rep-induced increase in the expression of inflammation-related factors, such as IL-6, TNF-α, monocyte chemotactic protein-1 (MCP-1), MMP-2 and MMP-9. However, LPS treatment alleviates the protective roles of ginsenoside Rb3 and activates the NF-κB pathway. Finally, the upstream factors of NF-κB were analyzed, including the Akt/Foxo3a and MAPK signaling pathways. We find that ginsenoside Rb3 pretreatment only decreases the phosphorylation of JNK induced by OGD-Rep injury, an indicator of the MAPK pathway. Importantly, an inhibitor of phospho-JNK, SP600125, protects against OGD-Rep induced apoptosis and inhibited NF-κB signaling pathway, similar to the roles of ginsenoside Rb3. Taken together, our results demonstrate that the protective effect of ginsenoside Rb3 on the OGD-Rep injury is attributed to the inhibition of JNK-mediated NF-κB activation, suggesting that ginsenoside Rb3 has the potential to serve as a novel therapeutic agent for myocardial I/R injury.

## Introduction

Ischemic myocardial disease is a complicated heart disorder worldwide, of which the main and most common cause is coronary atherosclerosis caused by stenosis or occlusion [1]. It is characterized by the decreased blood flow to the myocardium, resulting in the deficient supply of glucose, oxygen and other nutrients that are essential to generate energy. The perfusion of the ischemic myocardium is a crucial therapeutic strategy to alleviate ischemic symptoms [2]. However, injury occurs in the myocardium after perfusion, called “perfusion-injury” [3]. Myocardial ischemia-perfusion (I/R) injuries are complex pathophysiological processes, during which the reactive oxygen species (ROS) are generated, the calcium are overloaded and the mitochondrial permeability transition (MPT) pore opens, resulting in cell death or apoptosis [4]. Many pro-inflammatory cytokines are also released during the processes of I/R injury, such as tumor necrosis factor-α (TNF-α) [5]. Previous studies have identified that matrix metalloproteinase (MMP) release contributes to the myocardial dysfunctions, such as the release of MMP-2 [6] and MMP-9 [7]. Therefore, the inhibition of MMPs or pro-inflammatory cytokines may be novel therapeutic strategy for myocardial I/R injury.

NF-κB is a nuclear transcription factor that can regulate the gene expression critical to the apoptosis and inflammation during various diseases, including ischemic pathology [8]. In its inactive form, NF-κB is sequestered in the cytoplasm, where it is bound by the IκB family proteins including IκB-α. Once NF-κB is activated by a stimulus, IκB-α is phosphorylated by IKK followed by degradation, resulting in the translocation of NF-κB subunits from the cytoplasm to the nucleus. Previous studies have shown that the NF-κB subunit p65 is associated with I/R injury in a liver model via the upregulation of inflammation [9]. Moreover, the components of the mitogen-activated protein kinase (MAPK) signaling pathway also participate in inflammation [10], [11], which are validated to be upstream factors of NF-κB, including p38 MAPK, extracellular signal-regulated kinase (ERK) and c-Jun NH(2)-terminal kinase (JNK).

Previous studies demonstrate that some inhibitors of the inflammatory cytokines or NF-κB molecules can alleviate the pathological features induced by I/R injury [12]. In addition to the molecular inhibitors, a large body of evidence suggests that some herbs play important roles in various diseases. For instance, a previous study has demonstrated that ginsenoside Rb3 exerts a neuronal protective effect on the in vitro I/R injury model by inhibiting cell apoptosis and inflammatory cytokines [13]. However, the underlying mechanism of ginsenoside Rb3 in myocardial I/R injury remains poorly understood. In this study, we used oxygen and glucose deprivation followed by reperfusion (OGD-Rep) to simulate myocardial I/R injury in vitro in mouse H9c2 cells, and investigated the roles and regulation mechanism of ginsenoside Rb3 in OGD-Rep injury. These data may elucidate the potential of ginsenoside Rb3 as a therapeutic strategy for myocardial I/R injury.

## Materials and Methods

### Cell culture

Mouse cardiac myoblast H9c2 cells were purchased from ATCC and cultured in Dulbecco’s Modified Eagle’s Medium (DMEM, Invitrogen, USA) supplemented with 10% (v/v) fetal bovine serum (FBS). The cells were maintained in a humidified incubator with 95% air and 5% CO_2_ at 37°C.

### Myocardial I/R injury model and ginsenoside Rb3 treatment

In this study, we used oxygen and glucose deprivation followed by reperfusion (OGD-Rep) to simulate myocardial I/R injury in vitro in mouse H9c2 cells. OGD was initiated according to the previously described procedures [14]. Briefly, the cells were seeded at a density of 3.0×10^5^ cells/well into 35 mm plates before the experiment. After being cultured for 24 h, the cell culture medium was replaced with glucose-free DMEM, and the cells were maintained at 37°C in an oxygen-free incubator (95% N_2_ and 5% CO_2_). After incubation for 4 h, the glucose content in the culture medium was adjusted to normal level (4.5 mg/mL) at 37°C atmosphere with 95% air and 5% CO_2_, and the cells were cultured for another 24 h as the model of OGD-reperfusion (OGD-Rep). Ginsenoside Rb3 (molecular weight: 1079.27) was extracted from the root of Panax ginseng with 98% purity and purchased from Sigma Company. It was diluted to different concentrations (2.0 µmol/L and 5.0 µmol/L) and added to the culture medium 24 h before the initiation of OGD. The cells were exposed to ginsenoside Rb3 throughout all OGD and OGD-Rep procedures. The cells cultured in normal medium at 37°C in a 95% air and 5% CO_2_ atmosphere were used as control.

### Cell apoptosis assay

Cell apoptosis was analyzed using the Annexin V-PE/7-AAD kit and flow cytometric analysis. Briefly, the cells treated with ginsenoside Rb3 followed by OGD-Rep were washed with ice-cold PBS. They were then centrifuged for 5 min at 500×g at 4°C. After centrifugation, the cell pellets were resuspended in ice-cold 1×Binding Buffer with a density of 10^7^ cells/ml. Subsequently, 10 µl of Annexin V-PE solution and 20 µl of 7-AAD viability Dye were added to 100 µl of this cell suspensions. The tubes were maintained on ice and incubated for 15 min in the dark. Finally, 400 µl of ice-cold 1×Binding Buffer was added into the tubes followed by flow cytometry analysis on a BD FACS Calibur Flow Cytometry System (Becton Dickson, USA) according to the manufacturer’s protocols. The cells with Annexin V-PE^+^/7-AAD^–^ were calculated to represent apoptotic cells.

### Analysis of ROS by detection of superoxide and peroxynitrite formation

Superoxide was detected using the ROS Fluorescent Probe-DHE (Dihydroethidium) (Vigorous Biotechnology, China) according to the manufacturer’s instruction. Briefly, the cells were incubated with 1 µM of a DHE probe for approximately 30 min at 37°C, followed by being observed under a fluorescence microscope. We also detected the formation of peroxynitrite by monitoring the nitrosylated tyrosine protien levels via immunostaining, using the anti-3-nitrotyrosine (3-NT) antibody (1∶1000, Abcam). Superoxide or 3-NT positive cells were imaged in six regions under 20×magnification using a fluorescence microscope.

### Enzyme-linked immunosorbent assay (ELISA)

The NF-κB p65 ELISA Kit (StressXpress from Assay Designs/Stressgen Bioreagents) was used to measure NF-κB activation according to the manufacturer’s instructions by partitioning a cytoplasmic and nuclear p65 pool and assaying the concentration of p65 in the respective cellular compartment. The ratio of the nuclear relative to cytoplasmic p65 was used as an index of NF-κB activation. The Fivephoton Biochemicals Nuclear Protein Isolation Kit (Part NPI-1) was employed to isolate the nuclear and cytoplasmic fractions for ELISA.

### Western blot assay

The cells were subjected to Western blot analysis to determine the expression of IκB-α, NF-κB p65, Akt, Foxo3a, JNK, ERK and p38. Briefly, cells were washed with 1×PBS and lysed with RIPA buffer (50 mM Tris-HCl pH 7.4, 150 mM NaCl, 1% NP-40, 0.5% sodium deoxycholate, 0.1% SDS) for 10 min on ice. The nuclear protein was isolated as described above. After centrifugation for 10 min at 10000×g at 4°C, the supernatant was collected for the Western blot assay. The protein concentration was determined using the BCA method. 30 µg of protein was loaded on a 10% SDS-PAGE gel. A mouse monoclonal antibody to phospho-IκB-α (phospho Ser32 + Ser36, 1∶500 dilution), rabbit polyclonal antibody to phospho-IκB-α (phospho Y305, 1∶500 dilution), mouse polyclonal antibody to IκB-α (1∶1000 dilution), rabbit polyclonal antibody to phospho-NF-κB p65 (phospho S276, 1∶1000 dilution), rabbit polyclonal antibody to phospho-NF-κB p65 (phospho S536, 1∶1000 dilution), rabbit polyclonal antibody to NF-κB p65 (1∶1000 dilution), rabbit polyclonal antibody to phospho-Akt (phospho S473, 1∶1000 dilution), rabbit polyclonal antibody to Akt (1∶1000 dilution), rabbit polyclonal antibody to phospho-Foxo3a (phospho S253, 1∶1000 dilution), goat monoclonal antibody to Foxo3a (1∶1000 dilution), rabbit polyclonal antibody to phospho-JNK1+JNK2 (phospho T183+ Y185, 1∶1000 dilution), rabbit polyclonal antibody to JNK1+JNK2 (1∶1000 dilution), rabbit polyclonal antibody to phospho-Erk1 (pT202/pY204, 1∶1000 dilution) + Erk2 (pT185/pY187, 1∶1000 dilution), rabbit polyclonal antibody to Erk1 + Erk2 (1∶1000 dilution), rabbit polyclonal antibody to phospho-p38 (phospho T180 + Y182, 1∶1000 dilution) and rabbit polyclonal antibody to p38 (1∶1000 dilution) were purchased from Abcam Company and used as primary antibodies. The secondary antibodies were goat anti-rabbit or anti-mouse IgG conjugated with HRP (horseradish peroxidase) at a dilution of 1∶3000. The bound antibodies were detected with an ECL Plus western blotting Detection system (GE Healthcare) and the chemiluminiscent signals were detected with high-performance chemiluminescence film.

### Immunofluorescent microscopy

The treated cells were seeded at a density of 4000 cells/well into 14-well chambers. Once adhesive to the chambers, the cells were washed with PBS, fixed with 4% paraformaldehyde, and permeabilized with 0.5% Triton X-100. The cells were then blocked in 10% donkey serum for 1 h. A rabbit polyclonal antibody to NF-κB p65 and mouse monoclonal antibody to smooth muscle heavy chain (MHC) were used as the primary antibodies. The cells were incubated overnight at 4°C with primary antibodies in 1% donkey serum, and stained for 1.5 h with fluorescein isothiocyanate or PeCy5-conjugated secondary antibodies. MHC is a novel biomarker used to analyze the myocardial cell membrane and was employed as a control. After incubation with the secondary antibody, the cells were washed and stained with 4,6-diamino-2-phenyl indole (DAPI) for 5 min at room temperature. Finally, the slides were fixed and imaged with the fluorescent microscopy.

### Electrophoretic mobility shift assay (EMSA)

The cells treated with ginsenoside Rb3 followed by OGD-Rep were analyzed for NF-κB activity by electrophoretic mobility shift assay (EMSA). The double-stranded probed used to analyze the NF-κB binding sites (Santa Cruz Biotechnology) were: 5′-AGTTGAGGGGACTTTCCCAGGC-3′ and 3′-TCAACTCCCCTGAAAGGGTCCG-5′, which were labeled with [γ-32P] ATP according to the manufacturer’s protocols. The nuclear protein was isolated using the Fivephoton Biochemicals Nuclear Protein Isolation Kit (Part NPI-1). The EMSA was then performed using Pierce EMSA kit following the manufacturer’s instructions. The probe and nuclear protein complexes were subjected to non-denaturing 6% PAGE electrophoresis gel. Finally, the gels were vacuum-dried and exposed to an X-ray film at −70°C. The relative intensity of autoradiographic signals was performed using an enhanced chemiluminescence (Amersham, USA).

### RNA isolation and real-time PCR

Total RNA was extracted from the cells using Trizol (Invitrogen, USA) according to the manufacturer’s instructions. The RNA concentration was determined using a NanoDrop ND-2000 spectrophotometer (Thermo, USA). 500 ng of RNA was used for the cDNA synthesis using the PrimeScript 1st Strand cDNA Synthesis Kit (TaKaRa) according to the manufacturer’s instructions. Real-time PCR was performed using SYBR *Premix Ex Taq* (TaKaRa) according to the following conditions on an ABI 7300 Real-Time PCR machine: 95°C 3 min, followed by 40 cycles of 95°C 30 sec, 58°C 30 sec and 72°C 30 sec. β-actin was used as an internal control. The Real-time PCR primers were IL-6 sense: 5′CTGCGCAGCTTTAAGGAGTTC3′; anti-sense: 5′TCTGAGGTGCCCATGCTACA3′; TNF-α sense: 5′CCTGCCCCAATCCCTTTATT3′; anti-sense: 5′CCAATTCTCTTTTTGAGCCAGAA3′; MCP-1 sense: 5′CTCTCGCCTCCAGCATGAA3′; anti-sense: 5′GGGAATGAAGGTGGCTGCTA3′; MMP-2 sense: 5′GTGCCCAAGAATAGATGCTGACT3′; anti-sense: 5′TCTGAGGGTTGGTGGGATTG3′; MMP-9 sense: 5′GCGCTGGGCTTAGATCATTC3′; anti-sense: 5′AGGTGCCGGATGCCATT3′; β-actin sense: 5′CTCCATCCTGGCCTCGCTGT3′; antisense: 5′GCTGTCACCTTCACCGTTCC3′.

### Statistical analysis

All data were acquired from three independent experiments and represented as the mean ± SD. Each group was tested in triplicate. The differences were analyzed using two-tailed Student’s t-test and p<0.05 was considered statistically significant.

## Results

### Ginsenoside Rb3 inhibits OGD-Rep-induced apoptosis in H9c2 cells

**Figure 1:**
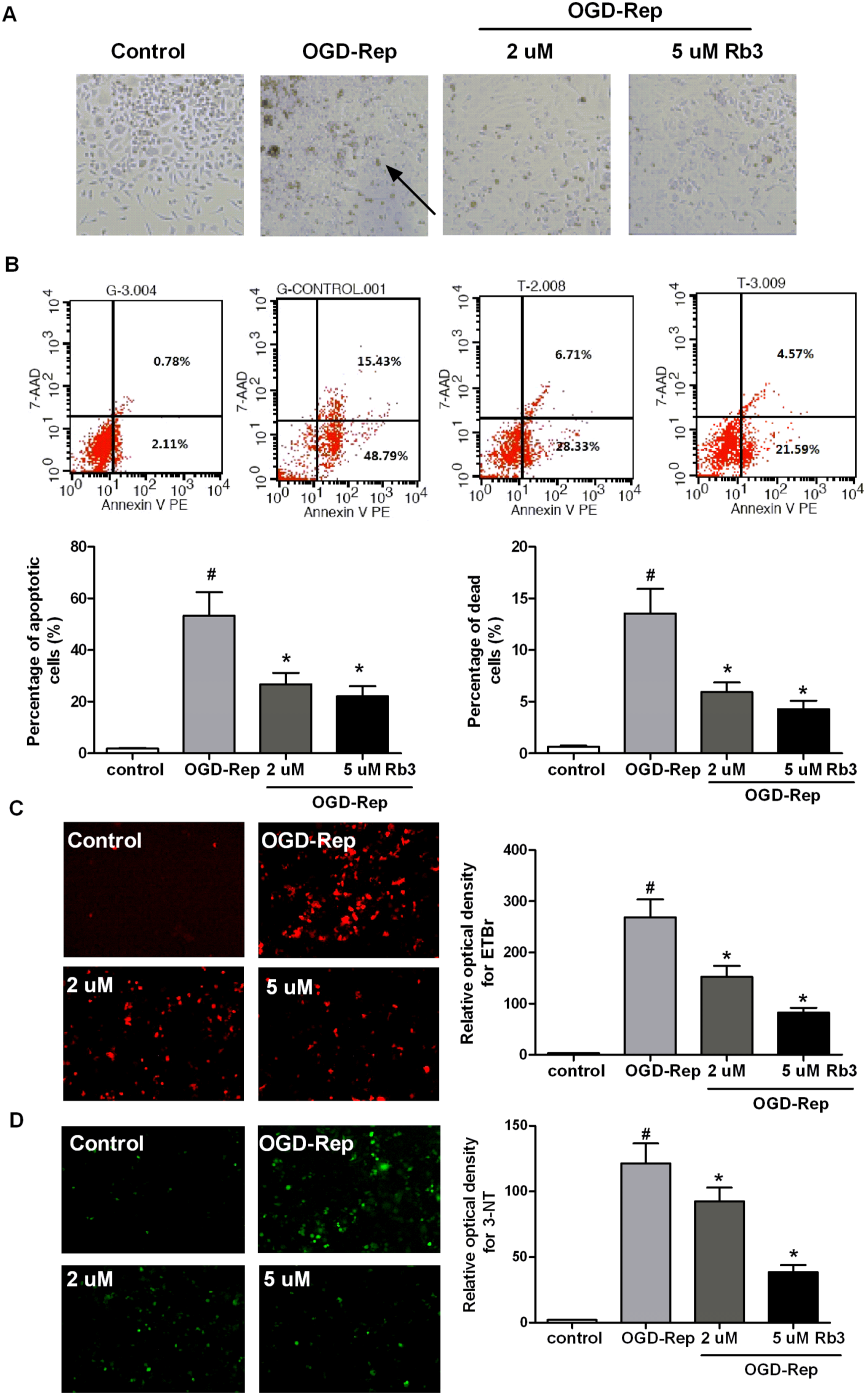
Ginsenoside Rb3 pretreatment protects against OGD-Rep-induced apoptosis in H9c2 cells. H9c2 cells were treated with ginsenoside Rb3 at different concentration (5 µM or 2 µM) followed by OGD-Rep. (A) cell images at 20×magnification under microscope. The arrow indicated the apoptotic cells. (B) Cell apoptosis was determined by Annexin V-PE/7-AAD assay. The cells stained with Annexin V-PE were apoptotic cells (the lower right corner), and the cells stained with 7-AAD were dead or in the late stages of apoptosis (the upper right corner). The graph represented the percentage of apoptotic and dead cells. Ginsenoside Rb3 decreased the cell apoptosis and death. (C) The fluorescent images after incubation with the DHE probe represented the superoxide levels (red). The graph was the quantitative measurement of the fluorescence. Ginsenoside Rb3 reduced the superoxide fluorescence. (D) The immunostaining image represented the formation of 3-NT (green). The graph represented the quantitative measurement of the formation of 3-NT. Ginsenoside Rb3 inhibited the 3-NT formation. The cells without OGD-Rep were used as control. All data were shown as the mean ± SD. ^#^P<0.05 vs. group without OGD-Rep. *P<0.05 vs. group treated with OGD-Rep alone. n = 3–4.

Because I/R injury results in cell apoptosis, we aimed to determine the roles of ginsenoside Rb3 in this process. The cells were treated with different concentration of ginsenoside Rb3 (2.0 µM and 5.0 µM) followed by OGD-Rep and subjected to apoptosis analysis via Annexin V-PE/7-AAD staining (Fig. 1). We found that OGD-Rep significantly increased the percentage of apoptotic cells from 2.11% to 48.79%, and ginsenoside Rb3 reduced the OGD-Rep-induced apoptosis by approximately 50%. To determine whether the inhibitory roles of ginsenoside Rb3 in apoptosis are related to ROS, we examined the effect of ginsenoside Rb3 on ROS levels. As shown in Fig. 1C and 1D, we found that OGD-Rep increased the production of superoxide and 3-NT, while ginsenoside Rb3 pretreatment reduced the increase in the superoxide and 3-NT production induced by OGD-Rep. These data suggest that ginsenoside Rb3 protects against OGD-Rep-induced apoptosis via the inhibition of ROS levels.

### Ginsenoside Rb3 inhibits OGD-Rep-induced NF-κB activation

**Figure 2:**
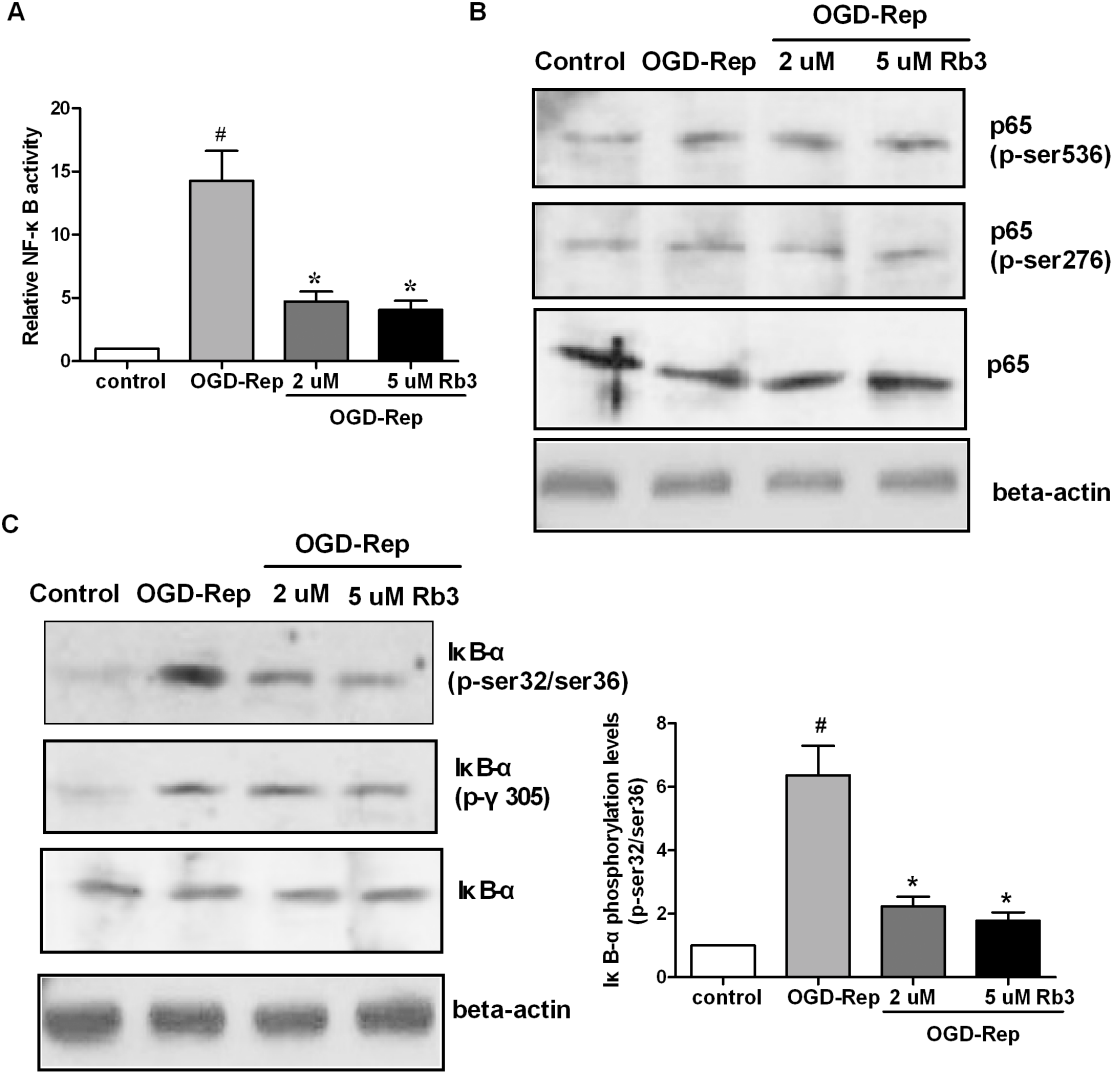
Ginsenoside Rb3 suppresses OGD-Rep-induced NF-κB activation in H9c2 cells. H9c2 cells were treated with ginsenoside Rb3 at different concentration (5 µM or 2 µM) followed by OGD-Rep. The cells were harvested and subjected to ELISA and Western blot assays. (A) The NF-κB activity was analyzed using a NF-κB p65 ELISA Kit, and the graph represented the ratio of the nuclear to the cytoplasmic p65 concentration. (B) The NF-κB p65 protein levels and NF-κB p65 phosphorylation were analyzed by Western blot assay. Beta-actin was used as an internal control. (C) The IκB-α protein levels and IκB-α phosphorylation were determined by Western blot assay, and beta-actin was used as an internal control. The graph represented the ratio of phosphorylated IκB-α to beta-actin. Ginsenoside Rb3 blocked OGD-Rep-induced increase in IκB-α phosphorylation levels. All data were shown as the mean ± SD. ^#^P<0.05 vs. group without OGD-Rep. *P<0.05 vs. group treated with OGD-Rep alone. n = 4–6.

Considering the key roles of NF-κB in I/R injury, we determine whether the protective roles of ginsenoside Rb3 against OGD-Rep-induced apoptosis are associated with the NF-κB pathway. First, we examined the NF-κB p65 protein levels in the cytoplasm and nucleus using ELISA and acquired the index of NF-κB activation. The results showed that OGD-Rep significantly induced the activation of NF-κB, and ginsenoside Rb3 (2.0 µM and 5.0 µM) suppressed the OGD-Rep-induced NF-κB activation by approximately 70% (Fig. 2A). Second, Western blot analysis showed that OGD-Rep increased the phosphorylation of NF-κB p65, while ginsenoside Rb3 pretreatment did not affect the OGD-Rep-induced increase in NF-κB p65 (Fig. 2B). Moreover, we found that OGD-Rep increased the phosphorylation of IκB-α, an indicator of NF-κB activation, and ginsenoside Rb3 blocked the increase in the phosphorylated IκB-α induced by OGD-Rep (Fig. 2C).

### Ginsenoside Rb3 inhibits OGD-Rep-induced nuclear translocation of NF-κB p65

**Figure 3:**
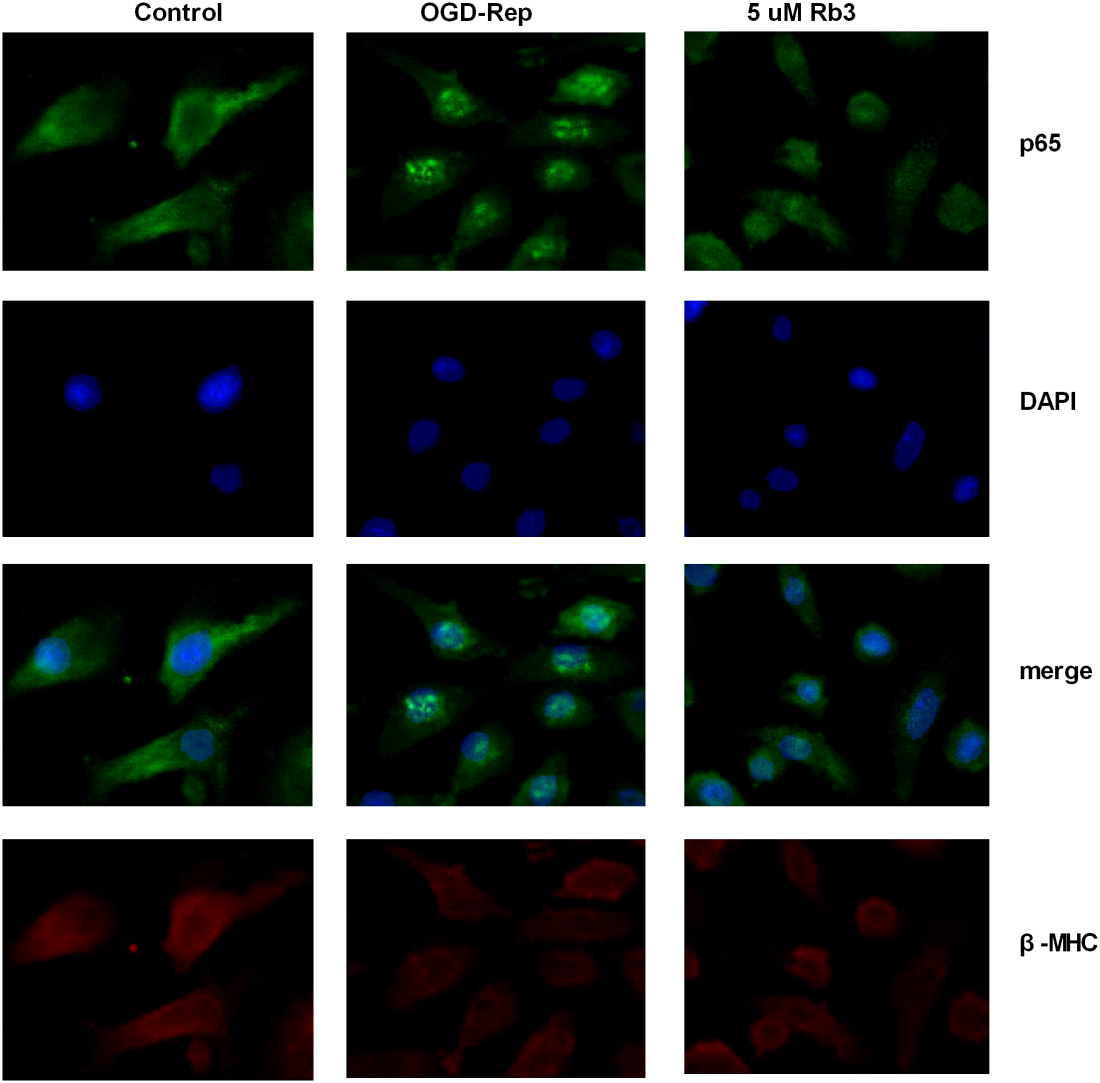
Ginsenoside Rb3 suppresses the nuclear translocation of NF-κB p65 induced by OGD-Rep in H9c2 cells. H9c2 cells were treated with 5 µM ginsenoside Rb3 followed by OGD-Rep. The cells were then subjected to an immunofluorescence assay to analyze the translocation of the NF-κB subunit p65. Ginsenoside Rb3 inhibited the nuclear translocation of p65 induced by OGD-Rep. The image was obtained at 20×magnification. Myosin heavy chain (MHC) was used as a marker of myocardial cells, and DAPI was used to stain nucleus. n = 6.

Once NF-κB is activated, the NF-κB subunit p65 is translocated to the nucleus and binds to its target genes to enact biological roles. Therefore, we used immunofluorescence to detect the positioning changes of NF-κB p65. As shown in Fig. 3, we found that OGD-Rep contributed to the nuclear translocation of p65, while ginsenoside Rb3 pretreatment inhibited the nuclear translocation induced by OGD-Rep.

### Ginsenoside Rb3 inhibits NF-κB binding activity induced by OGD-Rep

**Figure 4:**
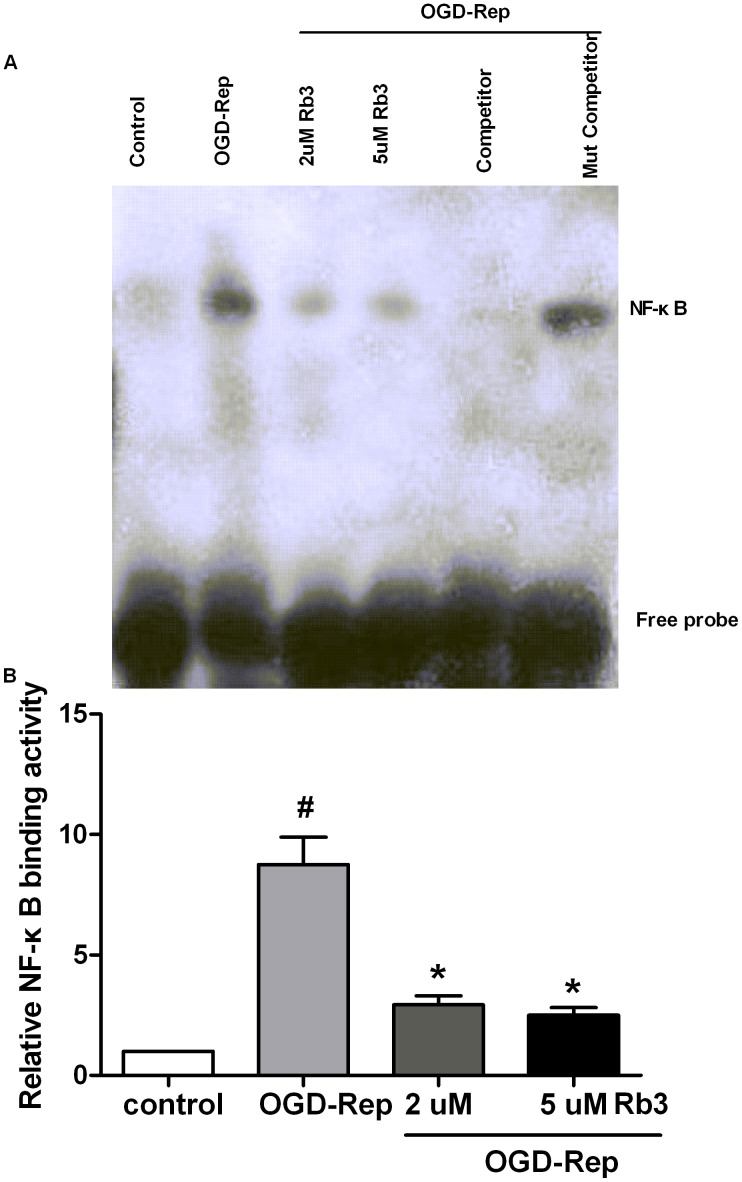
Ginsenoside Rb3 suppresses NF-κB binding activity induced by OGD-Rep in H9c2 cells. H9c2 cells were treated with ginsenoside Rb3 at different concentration (5 µM or 2 µM) followed by OGD-Rep. (A) The nuclear proteins were extracted and subjected to EMSA to analyze the NF-κB DNA binding activity. A competitor probe was used as a negative control. The cells treated with ginsenoside Rb3 showed a lower NF-κB DNA binding activity. (B) The graph represented the quantitative measurement of the NF-κB DNA binding intensity. All data were shown as the mean ± SD. ^#^P<0.05 vs. group without OGD-Rep. *P<0.05 vs. group treated with OGD-Rep alone. n = 4–6.

The above results suggested that ginsenoside Rb3 suppressed OGD-Rep-induced NF-κB activation. We next investigated the roles of ginsenoside Rb3 in NF-κB DNA binding activity by electrophoretic mobility shift assay (EMSA). As shown in Fig. 4, OGD-Rep significantly induced NF-κB binding activity. However, ginsenoside Rb3 pretreatment reduced the upregulation of NF-κB binding activity induced by OGD-Rep.

### Ginsenoside Rb3 inhibits simulative OGD-Rep-induced expression of IL-6, TNF-α, MCP-1, MMP-2 and MMP-9

**Figure 5:**
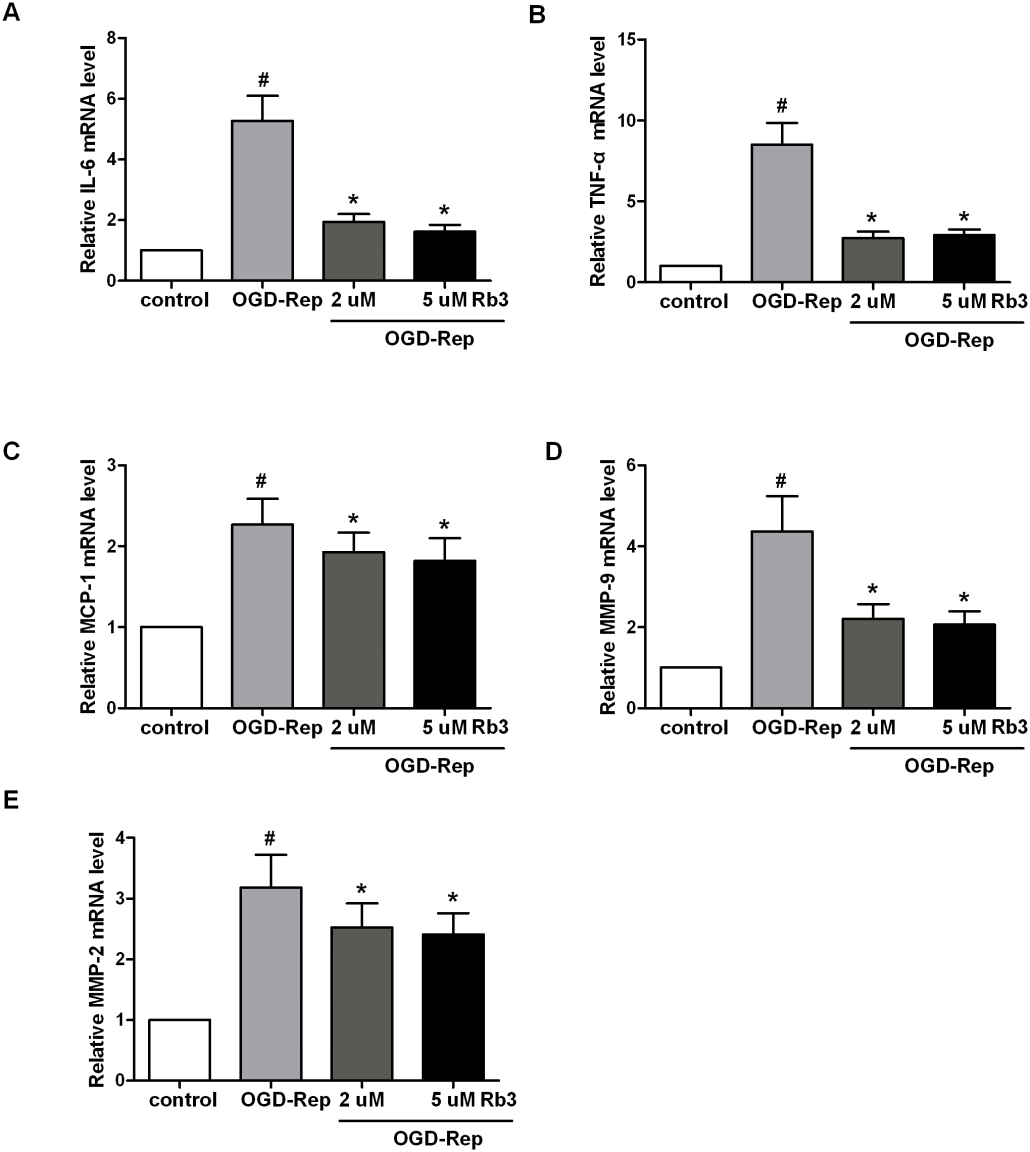
Ginsenoside Rb3 suppresses the expression of inflammation-related factors induced by OGD-Rep in H9c2 cells. H9c2 cells were treated with ginsenoside Rb3 at different concentration (5 µM or 2 µM) followed by OGD-Rep. The total RNA was isolated and subjected to real time PCR to analyze the products of pro-inflammatory cytokines, IL-6, TNF-α and MCP-1, as well as MMP-9 and MMP-2. Beta-actin was used as an internal control. All data were shown as the mean ± SD. ^#^P<0.05 vs. group without OGD-Rep. *P<0.05 vs. group treated with OGD-Rep alone. n = 4–6.

To investigate the roles of ginsenoside Rb3 in the inflammation induced by OGD-Rep, we observed the expression of various chemokines and pro-inflammatory cytokines. As shown in Fig. 5, we found that OGD-Rep significantly increased the expression of monocyte chemotactic protein 1 (MCP-1), IL-6 and TNF-α. Ginsenoside Rb3 pretreatment significantly suppressed the OGD-Rep-induced increase in the MCP-1, IL-6 and TNF-α mRNA levels.

Matrix metalloproteinases (MMPs) contribute to collagen degradation and the remodeling of the extracellular matrix after myocardial infarction [6]. In our study, we observed that OGD-Rep induced the mRNA expression of MMP-2 and MMP-9, while ginsenoside Rb3 significantly prevented OGD-Rep-induced expression of MMP-2 and MMP-9. Taken together, these data indicate that ginsenoside Rb3 suppresses OGD-Rep-induced inflammation.

### LPS-induced NF-κB activation suppresses the protective roles of ginsenoside Rb3 in OGD-Rep injury

**Figure 6:**
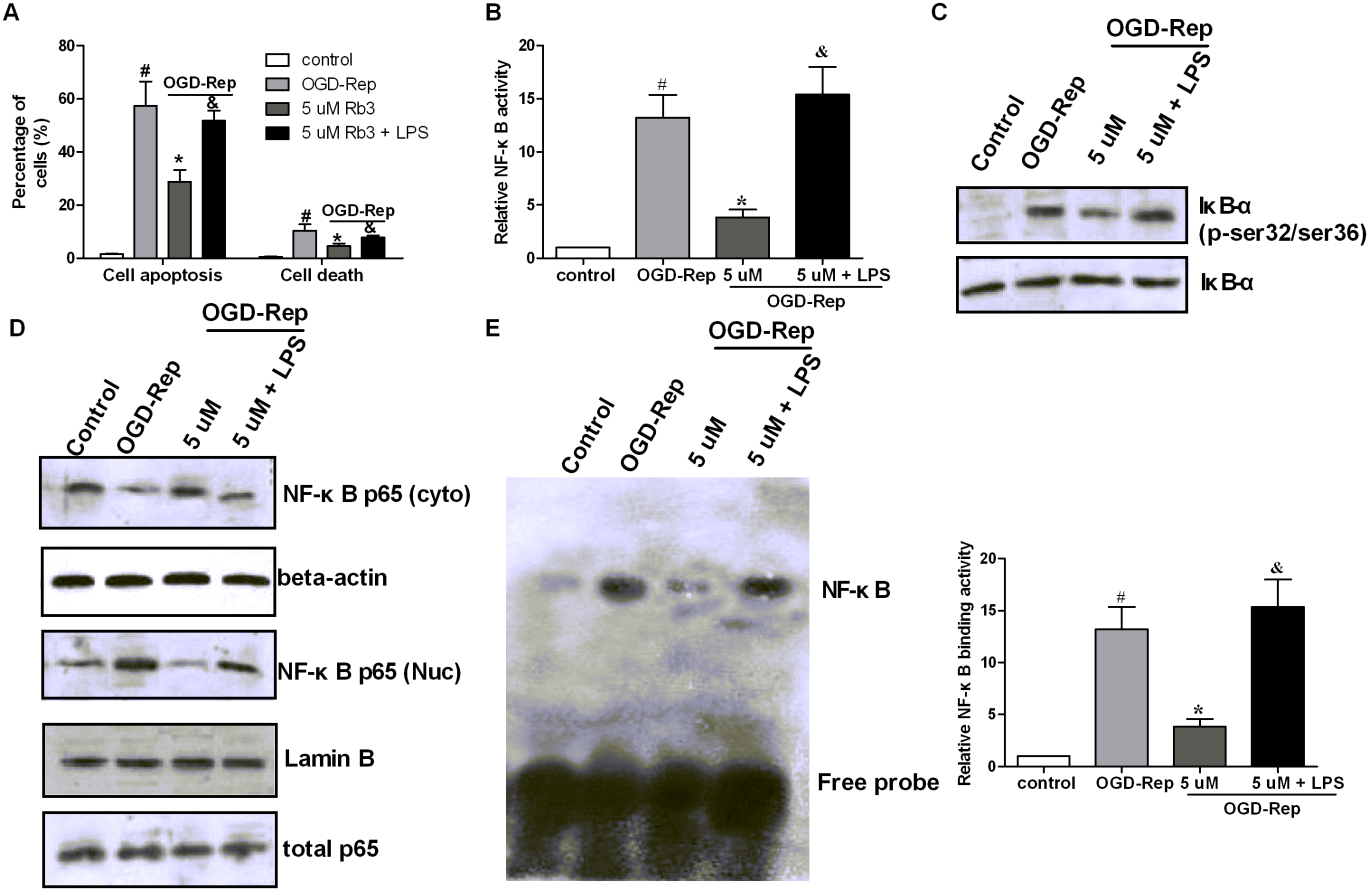
LPS-induced NF-κB activation alleviates the protective roles of ginsenoside Rb3. H9c2 cells were treated with 5 µM ginsenoside Rb3 and LPS followed by OGD-Rep, together with the controls. (A) The cell apoptosis was determined by Annexin V-PE/7-AAD assay. The graph represented the percentage of apoptotic and dead cells. LPS treatment increased the number of apoptotic and dead cells that was reduced by ginsenoside Rb3. (B) The NF-κB activity was analyzed using a NF-κB p65 ELISA Kit, and the graph represented the ratio of the nuclear to the cytoplasmic p65 concentration. LPS treatment increased the NF-κB activity that was reduced by ginsenoside Rb3. (C) The cell lysates were collected and subjected to Western blot assay to analyze the IκB-α phosphorylation and the total IκB-α protein levels. LPS treatment increased the IκB-α phosphorylation at ser32/ser36 that was reduced by ginsenoside Rb3. (D) The nuclear and cytoplasmic proteins were isolated and subjected to Western blot assay to analyze the protein levels of p65 in the nucleus and cytoplasm. Lamin B was used as the internal control to normalize the nuclear p65, and beta-actin was used as the internal control to normalize the cytoplasmic p65. LPS treatment increased the nuclear p65 levels and recued the cytoplasmic p65 levels compared to the cells treated with ginsenoside Rb3. (E) The nuclear proteins were extracted and the NF-κB DNA binding activity was analyzed by EMSA. The image represented the EMSA results, and the graph represented the relative NF-κB binding activity. LPS treatment increased NF-κB binding activity, compared to the cells treated with ginsenoside Rb3. The cells without OGD-Rep were used as a control. All data were shown as the mean ± SD. ^#^P<0.05 vs. group without OGD-Rep. *P<0.05 vs. group treated with OGD-Rep alone. &P<0.05 vs. group treated with ginsenoside Rb3 followed by OGD-Rep. n = 4–6.

The above results indicate that ginsenoside Rb3 might protect the cells from OGD-Rep injury via the suppression of the NF-κB signaling pathway. To validate this hypothesis, we used lipopolysaccharide (LPS) to activate NF-κB and observed the roles of ginsenoside Rb3 in OGD-Rep injury and the NF-κB signaling pathway. As shown in Fig. 6A, we found that LPS treatment increased the numbers of apoptotic and dead cells, which were reduced by ginsenoside Rb3. After testing the NF-κB signaling pathway, we discovered that the NF-κB activity, phosphorylation levels of IκB-α and NF-κB binding activities of cells treated with LPS and ginsenoside Rb3 were higher than those of cells with ginsenoside Rb3. Consistently, the translocation of the NF-κB subunit p65 from the cytoplasm to nucleus was increased after LPS treatment (Fig. 6B–6E). Taken together, these data demonstrate that the inhibition of NF-κB activation participates in the protective effect of ginsenoside Rb3 in OGD-Rep injury.

### Ginsenoside Rb3 inhibits OGD-Rep-induced activation of the MAPK pathway

**Figure 7:**
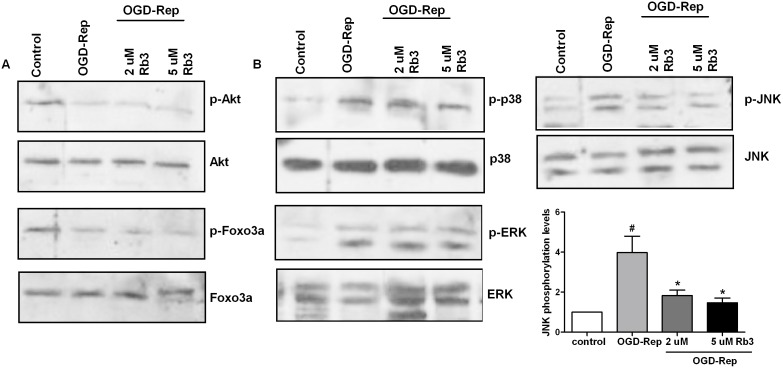
The effect of ginsenoside Rb3 on the upstream of NF-κB pathway in OGD-Rep-induced H9c2 cells. H9c2 cells were treated with ginsenoside Rb3 at different concentrations (5 µM or 2 µM) followed by OGD-Rep. The cells were harvested and subjected to Western blot assay to analyze the Akt protein levels, Akt phosphorylation, Foxo3a protein levels and Foxo3a phosphorylation (A), as well as JNK, JNK phosphorylation, ERK, ERK phosphorylation, p38 and p38 phosphorylation (B). The graph represented the quantitative measurement of the JNK phosphorylation and JNK protein levels. Beta-actin was used as an internal control. All data were shown as the mean ± SD. ^#^P<0.05 vs. group without OGD-Rep. *P<0.05 vs. group treated with OGD-Rep alone. n = 3–4.

To further discuss the roles of the upstream effectors of NF-κB signaling pathway in the protective process of ginsenoside Rb3 against OGD-Rep injury, we detected the expression of the Akt/Foxo3a and MAPK family members p38 MAPK, ERK and JNK. As shown in Fig. 7A, we observed that OGD-Rep suppressed the phosphorylation levels of Akt and Foxo3a, but ginsenoside Rb3 did not affect the reduction in the phosphorylated Akt and Foxo3a due to OGD-Rep. When examining the MAPK pathway, we discovered that OGD-Rep significantly induced the phosphorylation levels of JNK, ERK and p38. Pre-treatment with ginsenoside Rb3 inhibited the increase in phosphorylated JNK. However, ginsenoside Rb3 did not affect the phosphorylation levels of ERK and p38 induced by OGD-rep (Fig. 7B). These results suggest that ginsenoside Rb3 inhibits the OGD-Rep-induced NF-κB activation at least via the inhibition of JNK.

### Inhibition of phospho-JNK phenocopies the effect of ginsenoside Rb3

**Figure 8:**
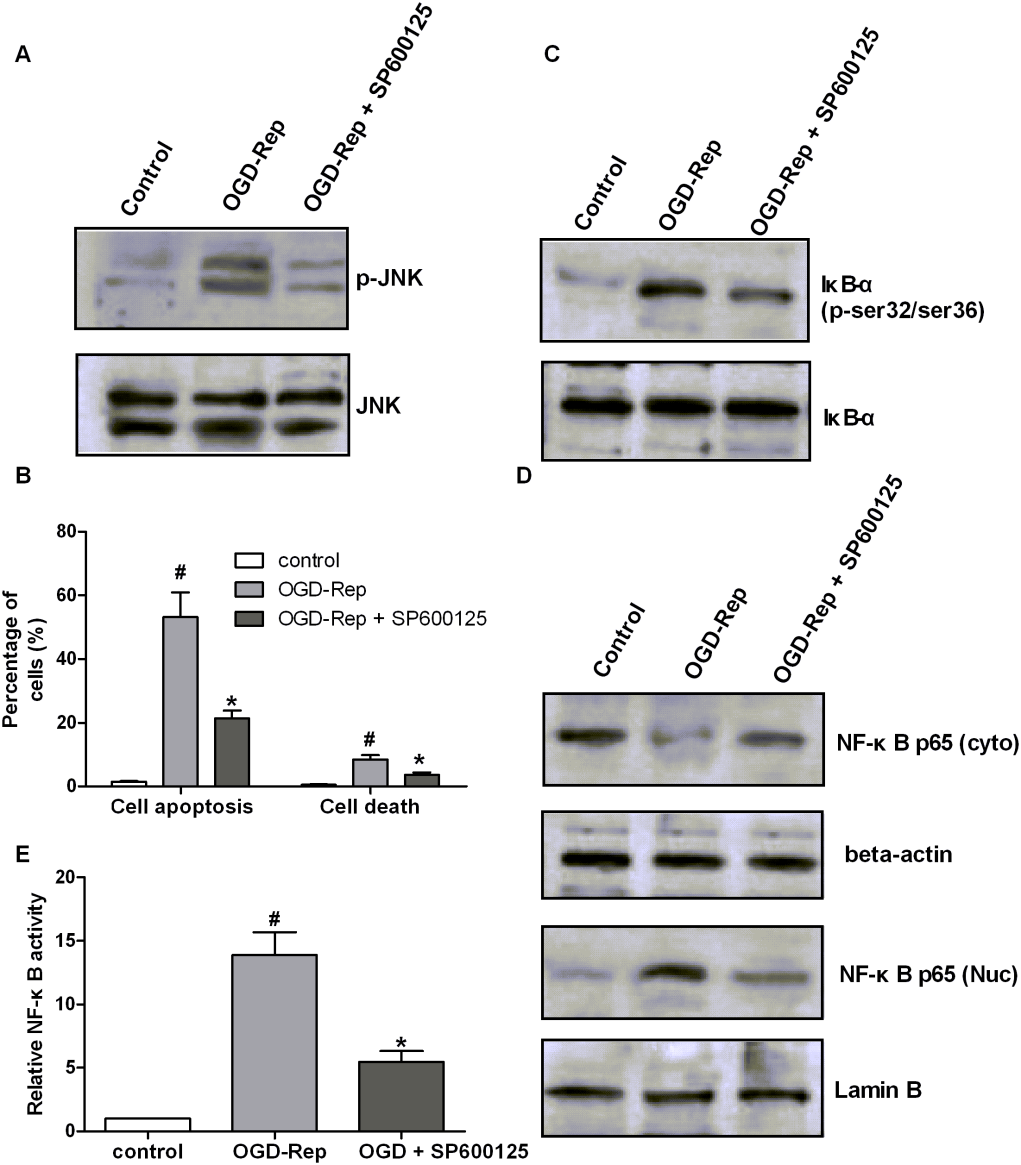
Inhibition of JNK phosphorylation phenocopies the effect of ginsenoside Rb3. H9c2 cells were treated with a JNK inhibitor, SP600125, followed by OGD-Rep. (A) Cell lysates were collected and subjected to Western blot assay to analyze the JNK phosphorylation and JNK protein levels. (B) The cell apoptosis was determined by Annexin V-PE/7-AAD assay. The graph represented the percentage of apoptotic and dead cells. SP600125 decreased the cell apoptosis and cell death that was induced by OGD-Rep. (C) The cell lysates were collected and subjected to Western blot assay to analyze the phosphorylation of IκB-α and total IκB-α protein. SP600125 decreased the IκB-α phosphorylation at ser32/ser36 sites that was induced by OGD-Rep. (D) The nuclear and cytoplasmic proteins were isolated and sunjected to Western blot assay to analyze the protein levels of p65 in the nucleus and cytoplasma. Lamin B was used as an internal control to normalize the nuclear p65, and beta-actin was used as an internal control to normalize the cytoplasmic p65. SP600125 decreased nuclear p65 levels and increased cytoplasmic p65 levels compared to the cells with OGD-Rep. (E) The NF-κB activity was analyzed using a NF-κB p65 ELISA Kit, and the graph represented the ratio of the nuclear to cytoplasmic p65 concentration. All data were shown as the mean ± SD. ^#^P<0.05 vs. group without OGD-Rep. *P<0.05 vs. group treated with OGD-Rep alone. n = 3–6.

We next investigate whether the JNK pathway is involved in the protective roles of ginsenoside Rb3 in OGD-Rep injury. A phospho-JNK inhibitor SP600125 was used and the phosphorylation levels of JNK were confirmed by Western blot (Fig. 8A). The cell apoptosis assay showed that SP600125 decreased the cell apoptosis and cell death induced by OGD-Rep (Fig. 8B). The NF-κB activation indicated that SP600125 inhibited IκB-α phosphorylation, reduced the nuclear p65 protein levels and increased the cytoplasmic p65 levels, while decreasing the NF-κB activity (Fig. 8C–8E). The results indicate that the inhibition of phospho-JNK protects the cells from apoptosis and suppresses the activation of the NF-κB pathway, similar to the effect of ginsenoside Rb3.

## Discussion

Although perfusion is a definitive treatment method for ischemic myocardial disease, injury induced by ischemia-perfusion (I/R) has become a complicated obstacle for effective therapy. Therefore, the mechanism underlying I/R injury has becomes a crucial focus of cardiovascular research. I/R injury has been validated to induce the release of many cytokines and chemokines, leading to cell apoptosis and death [15]–[18]. These reports were validated in our study, during which we found that OGD-Rep, a model of I/R injury, induced cardiac myoblast H9c2 cell apoptosis and ROS formation. Our study also showed that the levels of certain pro-inflammation cytokines and chemokines, such as IL-6, MCP-1, TNF-α, MMP-2 and MMP-9, were increased in OGD-Rep.

Ginsenosides, which are unique compounds of the Panax species, are under basic and clinical research to investigate their potential uses in medicine [19]. Studies have demonstrated that the components of ginsenosides play anti-inflammatory and anti-oxidant roles. For example, ginsenoside Rg1 exerts neuroprotective roles against exogenous hydrogen peroxide (H_2_O_2_)-induced oxidative stress via the inhibition of the NF-κB pathway and its upstream effectors, such as Akt and ERK [20]. Ginsenoside Rd has been validated to suppress myocardial I/R-induced cell apoptosis by decreasing ROS formation via the activation of the Akt-GSK3β pathway [21]. In agreement with the validated reports, our study demonstrated that ginsenoside Rb3 inhibited the H9c2 cell apoptosis and ROS formation induced by OGD-Rep. In addition, ginsenoside Rb3 decreased IκB-α phosphorylation, the translocation of NF-κB p65 to nucleus and the NF-κB DNA binding activity. It also decreased JNK phosphorylation, which is upstream of the upstream of NF-κB.

NF-κB is an important transcription factor involved in the process of inflammation and apoptosis induced by I/R injury. Sabarinathan Ramachandran *et.al* validate that the NF-κB subunit p65 is activated during I/R injury in the rat steatotic liver, with inflammation and necrosis [9]. Accordingly, the suppression of NF-κB by its inhibitor BAY 11-7802 or the inhibition of IκB phosphorylation has been validated to reduce the inflammation and apoptosis induced by myocardial or cerebral I/R injury [12], [22], [23]. With respect to the inflammatory mechanism of NF-κB, a large body of evidence has demonstrated that the NF-κB pathway is associated with the release of pro-inflammatory cytokines and chemokines. NF-κB activation has been shown to contribute to the expression of chemokine CCL2, also known as MCP-1, in human astrocytes [24]. Inhibiting nuclear translocation of NF-κB alleviates the production of MCP-1 during chronic inflammation [25]. In addition, the TNF-α-induced secretion of inflammatory cytokines, i.e., IL-6, is mediated by NF-κB signaling, and the inhibition of NF-κB reduces the expression of IL-6 [26]. Consistently, NF-κB activation upregulates the expression of IL-6 during multiple myeloma [27]. In addition to the induction of pro-inflammatory factors, NF-κB also increases the secretion of MMP-2 and MMP-9 [28], whose levels are increased in the reperfused myocardium after ischemia. Furthermore, these proteins play important roles in the degradation of the extracellular matrix and inflammatory development [6], [28], [29]. Consistent with the data in the previous reports, our results showed that NF-κB was activated in OGD-Rep injury and that the levels of the targets of NF-κB, i.e., inflammation-related genes such as MCP-1, IL-6, TNF-α, MMP-9 and MMP-2, were increased. To further validate the role of the inactivation of NF-κB in the protective roles of ginsenoside Rb3 in OGD-Rep, we used LPS [30] to activate NF-κB. The results showed that LPS treatment neutralized the inhibitory effect of Rb3 on cell apoptosis and death and increased the NF-κB signaling pathway activity that was suppressed by Rb3. The results further validate that Rb3 protects H9c2 cell from OGD-Rep via the inhibition of the NF-κB signaling pathway.

Several signaling pathways regulate the upstream of NF-κB, such as the MAPK and PI3K/Akt-related signaling pathways. PI3K/Akt phosphorylation contributes to IκB-α phosphorylation, leading to NF-κB activation [31]. In myocardial I/R injury, white wine exerts cardioprotective roles via the PI3K/Akt/FOXO3a/e-NOS/NF-κB survival pathway [32]. It is reported that MAPK, especially JNK, induces NF-κB activation at high glucose condition [33]. In addition to JNK, another two members of the MAPK family are also capable of contributing to NF-κB activity. [6]-gingerol suppresses pancreatic cancer metastasis via the inhibition of NF-κB induced by ERK [34]. Moreover, p38-MAPK is involved in the benzoquinone-mediated activation of NF-κB [35]. Although the levels of p38 and ERK phosphorylation were increased after OGD-Rep injury in our study, they did not change after ginsenoside Rb3 treatment. Our study also showed that ginsenoside Rb3 did not affect the levels of phospho-Akt/Foxo3a, which were reduced by OGD-Rep. However, our results showed that only phospho-JNK was increased in response to OGD-Rep injury, and ginsenoside Rb3 reduced its phosphorylation, similar to the effects observed in the NF-κB pathway. The differing responses of the upstream effectors of the NF-κB to ginsenoside Rb3 may be because that p38, ERK and Akt/Foxo3a phosphorylation are not sensitive to ginsenoside Rb3, and the regulation of NF-κB pathway by its upstream factors may be tissue- or cell-specific. We also used an inhibitor of phospho-JNK, SP600125, to confirm its roles in OGD-Rep injury. We found that SP600125 inhibited the activation of the NF-κB pathway and cell apoptosis that were induced by OGD-Rep.

## Conclusions

Our study indicated that ginsenoside Rb3 regulated OGD-Rep injury via the following mechanism: 1) OGD-Rep contributes to the activation of JNK followed by the phosphorylation of IκB-α, which leads to NF-κB activation; 2) once NF-κB is activated, it translocates to nucleus and binds to the transcriptional region of the pro-inflammatory factors, such as TNF-α, IL-6, MCP-1, and MMP-9 and MMP-2, resulting in cell apoptosis and inflammation; 3) ginsenoside Rb3 pretreatment prevents the above signaling pathway (JNK-mediated NF-κB pathway), and results in the protective effects. Our data indicate that ginsenoside Rb3 exerts its protective roles partly via the inhibition of JNK-mediated NF-κB pathway, suggesting that ginsenoside Rb3 has therapeutic potential for myocardial I/R. However, future studies should focus on the identification of more novel molecules that mediate the regulation of ginsenoside Rb3 in NF-κB activation.
